# Estrogen regulation of myokines that enhance osteoclast differentiation and activity

**DOI:** 10.1038/s41598-022-19438-4

**Published:** 2022-09-23

**Authors:** Andrew Norton, Kathleen Thieu, Cory W. Baumann, Dawn A. Lowe, Kim C. Mansky

**Affiliations:** 1 Division of Orthodontics, Department of Developmental and Surgical Sciences, University of Minnesota School of Dentistry, 515 Delaware St SE, Minneapolis, MN 55455 USA; 2 Division of Periodontology, Department of Developmental and Surgical Sciences, University of Minnesota School of Dentistry, Minneapolis, MN 55455 USA; 3 Ohio Musculoskeletal and Neurological Institute (OMNI), Department of Biomedical Sciences, Ohio University, Athens, OH 45701 USA; 4 Divisions of Rehabilitation Science and Physical Therapy, Department of Rehabilitation Medicine, University of Minnesota Medical School, Minneapolis, MN 55455 USA

**Keywords:** Cell biology, Diseases

## Abstract

Osteoporosis and sarcopenia are maladies of aging that negatively affect more women than men. In recent years, it has become apparent that bone and muscle are coupled not only mechanically as muscle pulls on bone, but also at a higher level with myokines, biochemical and molecular signaling occurring between cells of the two tissues. However, how estrogen deficiency in females impacts the chemical crosstalk between bone and muscle cells is not understood. We hypothesize that changes in estrogen signaling alters myokine expression and intensifies bone loss in women. In our present study, we demonstrate that conditioned media from ovariectomized or skeletal muscle deficient in estrogen receptor α (ERα) expression enhances osteoclast differentiation and activity. Using a cytokine array, we identified myokines that have altered expressions in response to loss of estrogen signaling in muscle. Lastly, we demonstrate that conditional deletion of ERα in skeletal muscle results in osteopenia due to an increase in the osteoclast surface per bone surface. Our results suggest that estrogen signaling modulates expression of myokines that regulate osteoclast differentiation and activity.

## Introduction

Although skeletal muscle and bone are anatomically coupled, the relationship between them is more than a purely mechanical interaction. Both muscle and bone are endocrine organs which can secrete factors to regulate the function of nearby tissues. Evidence suggests that there is crosstalk, or biomolecular signaling, between muscle and bone which contributes to the maintenance and function of the muscle and bone unit^1,2^. Previous studies suggest that bone and muscle communicate by paracrine, and endocrine signaling and may reciprocally coordinate growth and response to injury^2^.

Muscle secretes cytokines, peptides and growth factors termed “myokines” that affect bone formation and resorption^1,3–5^. One of the most abundant myokines is myostatin (also known as growth differentiation factor 8, GCD-8) which negatively regulates muscle mass^4^. Myostatin also negatively regulates bone maintenance by inhibiting osteoblasts and activating osteoclasts^6,7^. In an in vitro study, myostatin was shown to activate expression of osteogenic factors SOST, a negative regulator of bone formation and RANKL, a positive regulator of bone resorption, in osteocytes^8^. Another study demonstrated that conditioned media from electrically stimulated muscles protects osteocytes from glucocorticoid-induced apoptosis^9^. Osteoblasts treated with conditioned media from C2C12 cells leads to inhibition of osteoblast differentiation partially through the activity of ciliary neurotrophic factor (CNTF)^10^. Lastly, it was shown that conditioned media from mechanically loaded myotubes increased osteoclast formation that was blocked by an interleukin 6 (IL-6) neutralizing antibody^11^. Together, these studies suggest that contracting muscle releases factors that regulate the activity of bone cells.

Aging is associated with both osteoporosis and sarcopenia. Sarcopenia is the age-associated, progressive loss of skeletal muscle mass and strength while osteoporosis is a disease that thins and weakens the bones^12,13^. After age 35, there are significant decreases in muscle mass, strength, and power and such decrements are associated with poor balance, thus increasing susceptibility to falls and fractures^14,15^. Sarcopenia is also known to be a significant predictor of hospitalization in older individuals^16^. Individuals with osteoporosis and sarcopenia have reduced quality of life, higher risk of falls, morbidity, and loss of autonomy. While both men and women can experience osteoporosis and sarcopenia, women experience more severe reductions in muscle and bone mass when compared to men^17^. Differences in severity of osteoporosis and sarcopenia between men and women is associated with the reduction in estrogen levels due to menopause^18,19^. It is unclear which occurs first, osteoporosis or sarcopenia, but apparent that osteoporosis and sarcopenia are co-morbidities. One theory is that muscle declines first, leading to less skeletal loading, and thereafter reduced bone mass. Another theory is that as osteocytes age, there is decreased ability for osteocytes to produce the necessary factors required for maintenance of skeletal muscle^1^. However, neither of these theories can fully explain mechanisms linking estrogen deficiency, osteoporosis, and sarcopenia.

We hypothesize that altered myokine expression mediated by lack of estrogen signaling in skeletal muscle partially underlies exacerbated bone loss observed in females. To determine if changes in estrogen signaling alters myokines, we demonstrate that muscle condition media from ovariectomized (OVX) animals stimulates osteoclast differentiation and activity. Because ovaries secrete two groups of sex hormones, progesterone, and estrogen, we also treated osteoclast cultures with muscle conditioned media from mice conditionally deleted for *estrogen receptor 1* (*Esr1)*, hereafter ERα-KO mice, to determine if estrogen signaling through the major estrogen receptor was driving the skeletal response. Like the OVX conditioned media, conditioned media from ERα-KO muscle stimulates osteoclast differentiation and activity. We also show that myokine expression was altered in response to disruption in estrogen signaling in muscle. Lastly, mice lacking expression of ERα in skeletal muscle have osteopenia due to an increase in osteoclast differentiation. This study uniquely demonstrates that changes in myokine expression based on estrogen status impacts osteoclasts and ultimately bone health.

## Results

**Figure 1:**
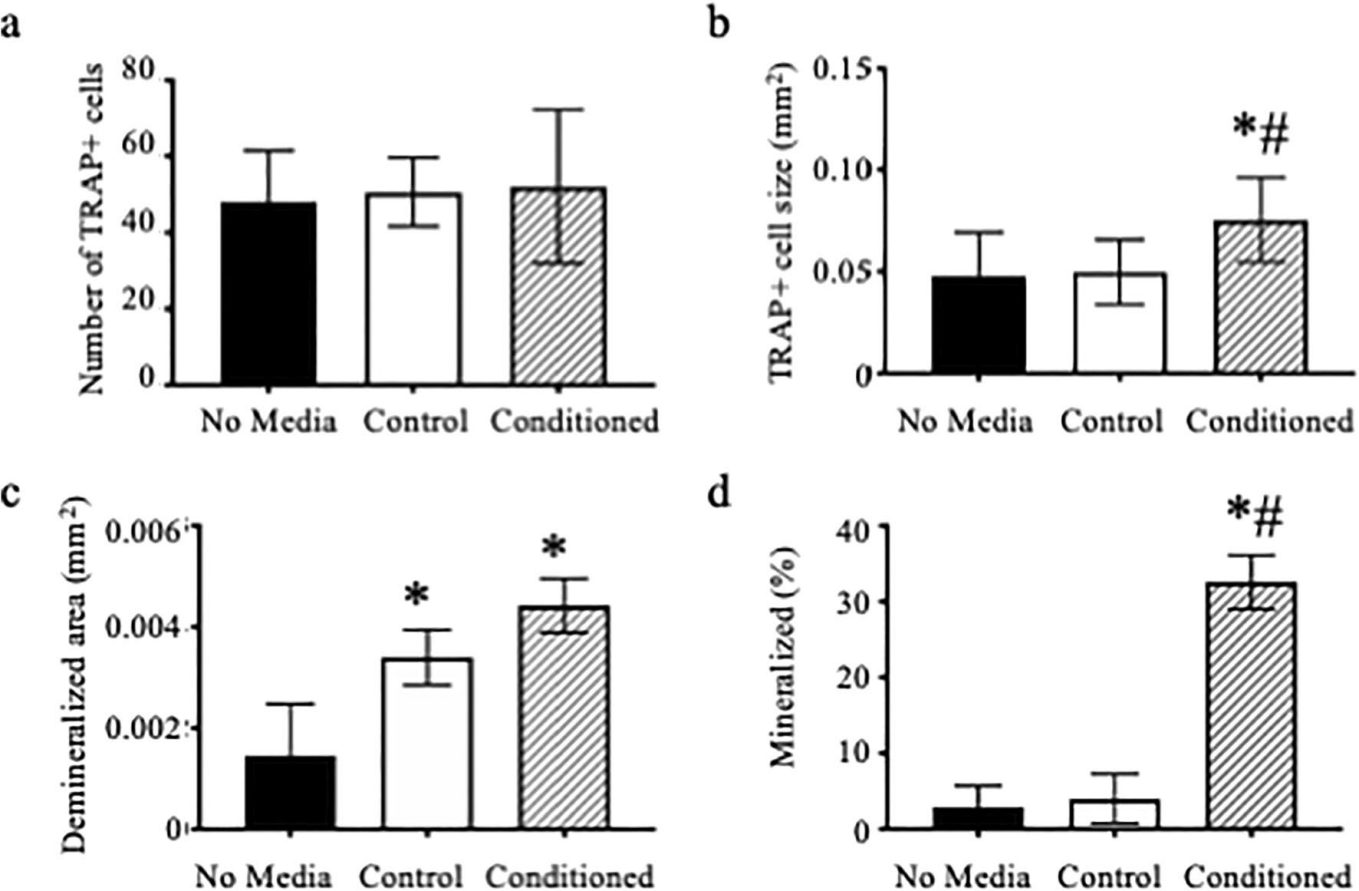
Muscle conditioned media enhances osteoclast and MC3T3 activity. (**A**–**C**) Bone marrow macrophages were treated with M-CSF and RANKL to induce osteoclast differentiation. At the time of RANKL addition, cultures were also given no media, Krebs media not exposed to muscle (Control) or muscle conditioned media, with the volume of the latter two conditions equal to 5% of the total media. (**A**) Average number and (**B**) average size of TRAP positive multinuclear cells and (**C**) average size of demineralized area. (**D**) MC3T3-E1 cells were grown to confluency and then induced to differentiate with the addition of ascorbic acid. Krebs (Control) or muscle conditioned media was given at the same time as ascorbic acid. Von Kossa assay was performed to determine percent mineralization. *Significantly different from No Media; ^#^Significantly different from Control.

Based on our preliminary experiments, we determined that 5% conditioned media provided a pronounced response in osteoclast and osteoblast differentiation. To determine if muscle expresses myokines that regulate differentiation, we collected media from extensor digitorum longus (EDL) muscles that completed an eccentric contraction injury protocol (producing conditioned media) and added that to osteoclast cultures throughout differentiation. We measured a significant increase in the size of TRAP multinuclear cells (Fig. 1B, *p* = 0.019), but did not measure any significant changes in the number of TRAP positive multinuclear cells (Fig. 1A, *p* = 0.918). We also treated osteoclasts cultured on calcium phosphate coated plates with muscle conditioned media and measured an increase in the average size of the demineralized area compared to cells that received no conditioned media (Fig. 1C, *p* = 0.05). To measure the effect of muscle myokines on the ability of osteoblasts to mineralize, we treated MC3T3-E1, an osteoblast cell line derived from mouse calvaria, with either Krebs buffer only (Control) or conditioned media throughout differentiation. MC3T3-E1 cells treated with muscle conditioned media had an 8-fold increase in the percent of mineralization area compared to cells treated with no media or media not exposed to muscle, i.e., control (Fig. 1D, *p* = 0.003).

**Figure 2:**
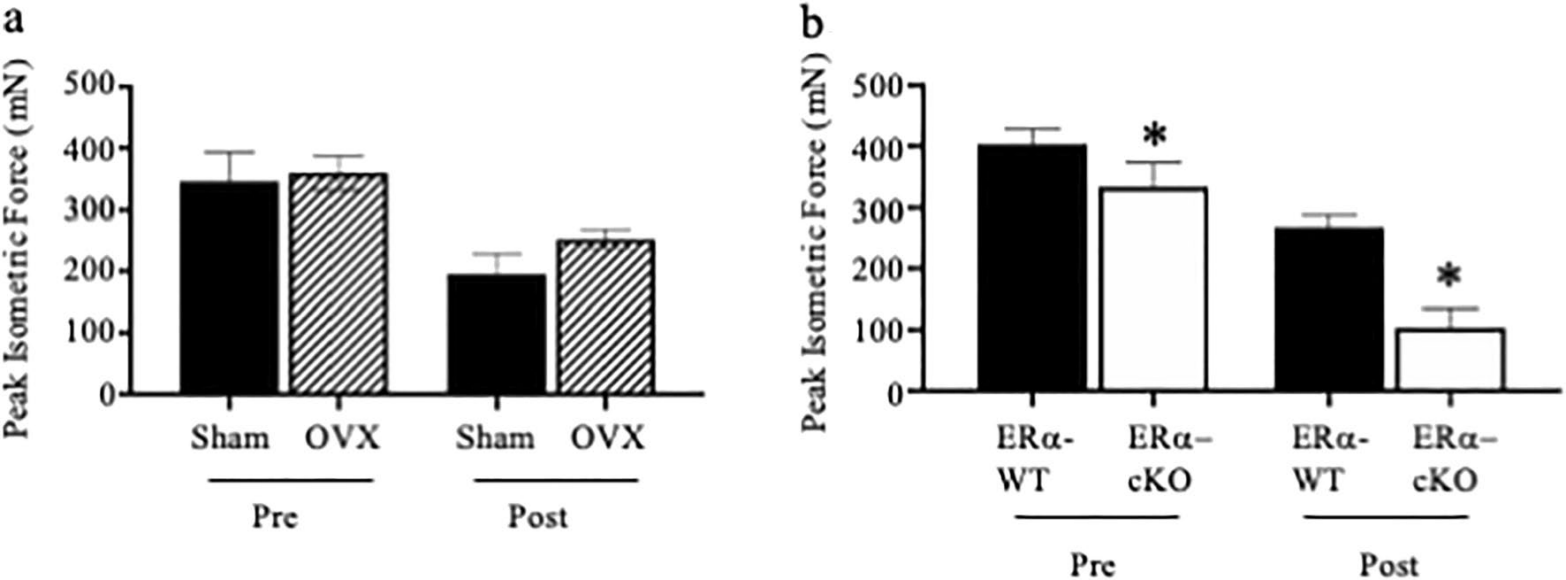
Peak isometric force is reduced following an eccentric contraction-induced injury protocol. (**A**) Peak isometric force of EDL muscles from Sham and OVX mice did not differ before (Pre) or after (Post) contractions. (**B**) Peak isometric force of EDL muscle with and without skeletal muscle estrogen receptor α (ERα) immediately before (Pre) and after (Post) a bout of 10 maximal, high force eccentric contractions was lower in ERα-cKO. Following the last contraction, the Krebs buffer surrounding each muscle was collected as the conditioned media and used in the in vitro bone cell assays. *Significantly different from ERα-cKO.

**Figure 3:**
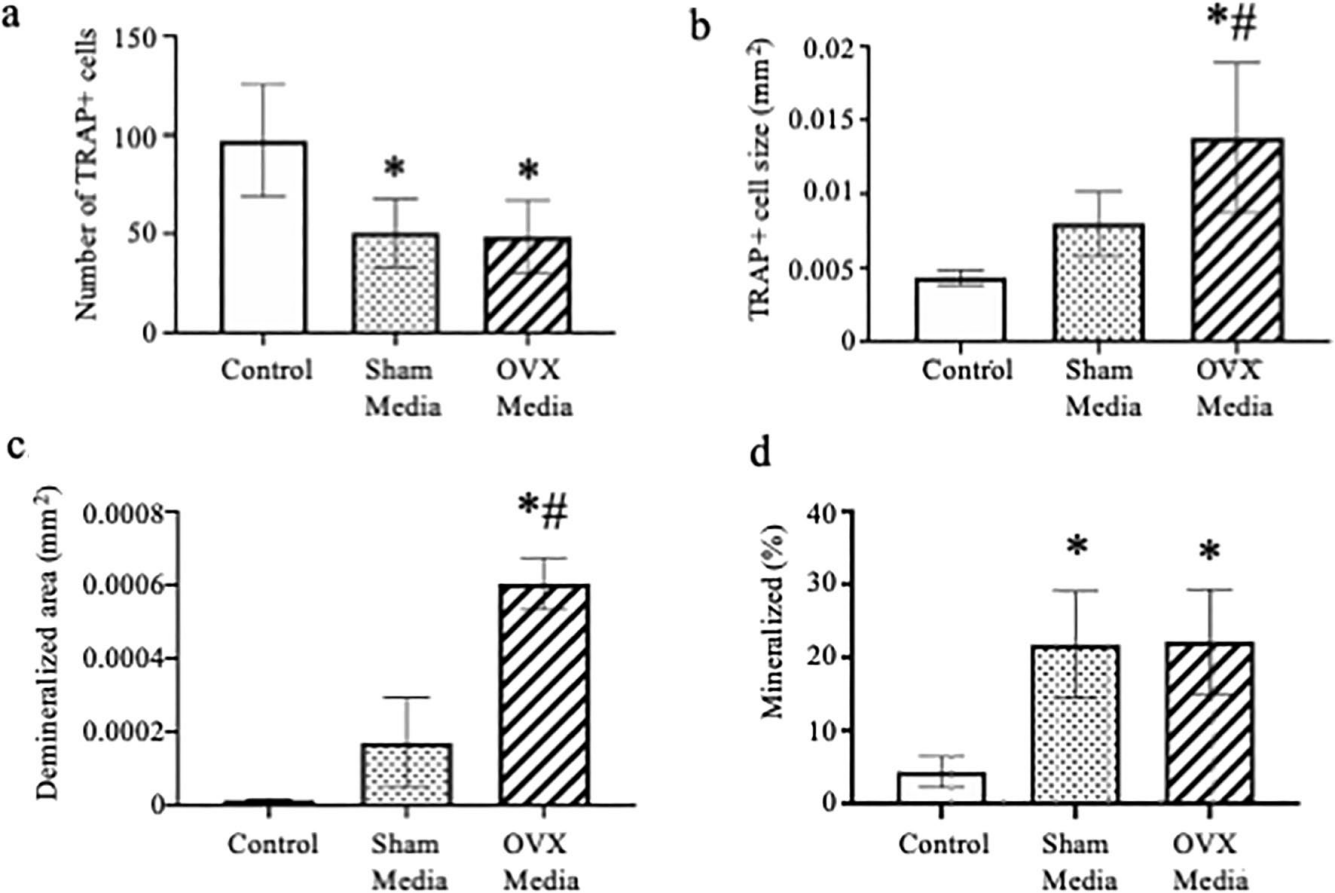
Muscle conditioned media from ovariectomized animals increases osteoclast size. (**A**–**C**) Bone marrow macrophages were treated with M-CSF and RANKL to induce osteoclast differentiation. At the time of RANKL addition cultures were also given 5% Krebs (Control) or muscle conditioned media from sham-operated or ovariectomized animals. (**A**) Average number and (**B**) average size of TRAP positive multinuclear cells and (**C**) average size of demineralized area. (**D**) MC3T3-E1 cells were grown to confluency and then induced to differentiate with the addition of ascorbic acid. Krebs (Control) or muscle conditioned media from sham-operated or ovariectomized animals was given at the same time as ascorbic acid. Von Kossa assay was done to measure percent mineralization of cells treated with 5% Krebs (control), Sham or OVX muscle conditioned media. *Significantly different from Control; ^#^Significantly different from Sham Media.

As we were able to measure an increase in osteoclast size and activity as well as the ability of osteoblasts to mineralize when treated with conditioned media from EDL muscles of healthy female mice, we next tested the effect of treating osteoclast cultures with conditioned media from muscles of Sham-operated and OVX mice. A bout of 10 high-force eccentric contractions by isolated EDL muscles caused peak isometric force to dro*p* ~ 40% in both Sham and OVX mice (Fig. 2A, *p* < 0.001). Conditioned media from both Sham and OVX mouse muscles resulted in a significant decrease in osteoclast number (Fig. 3A, *p* = 0.003) and a significant increase in osteoclast size in cells treated with OVX media (Fig. 3B, *p* = 0.001). Additionally, muscle conditioned media from OVX animals increased osteoclast size 1.5-fold over Sham muscle conditioned media (Fig. 3B, *p* = 0.043) and resulted in a significant increase in demineralization compared to osteoclasts treated with media from Sham animals (Fig. 3C, *p* ≤ 0.0001). When MC3T3-E1 cells were treated with Sham or OVX muscle conditioned media, mineralization increased fivefold compared to control; however, there was no significant difference in the response between Sham and OVX media (Fig. 3D, *p* = 0.983). EDL muscle mass was ~ 9% less in OVX than Sham mice (11.1 ± 0.6 and 12.1 ± 0.5 mg, respectively; *p* = 0.020) suggesting that even though the smaller OVX muscle had less volume to release myokines it imposed a relatively greater impact on osteoclast biology.

**Figure 4:**
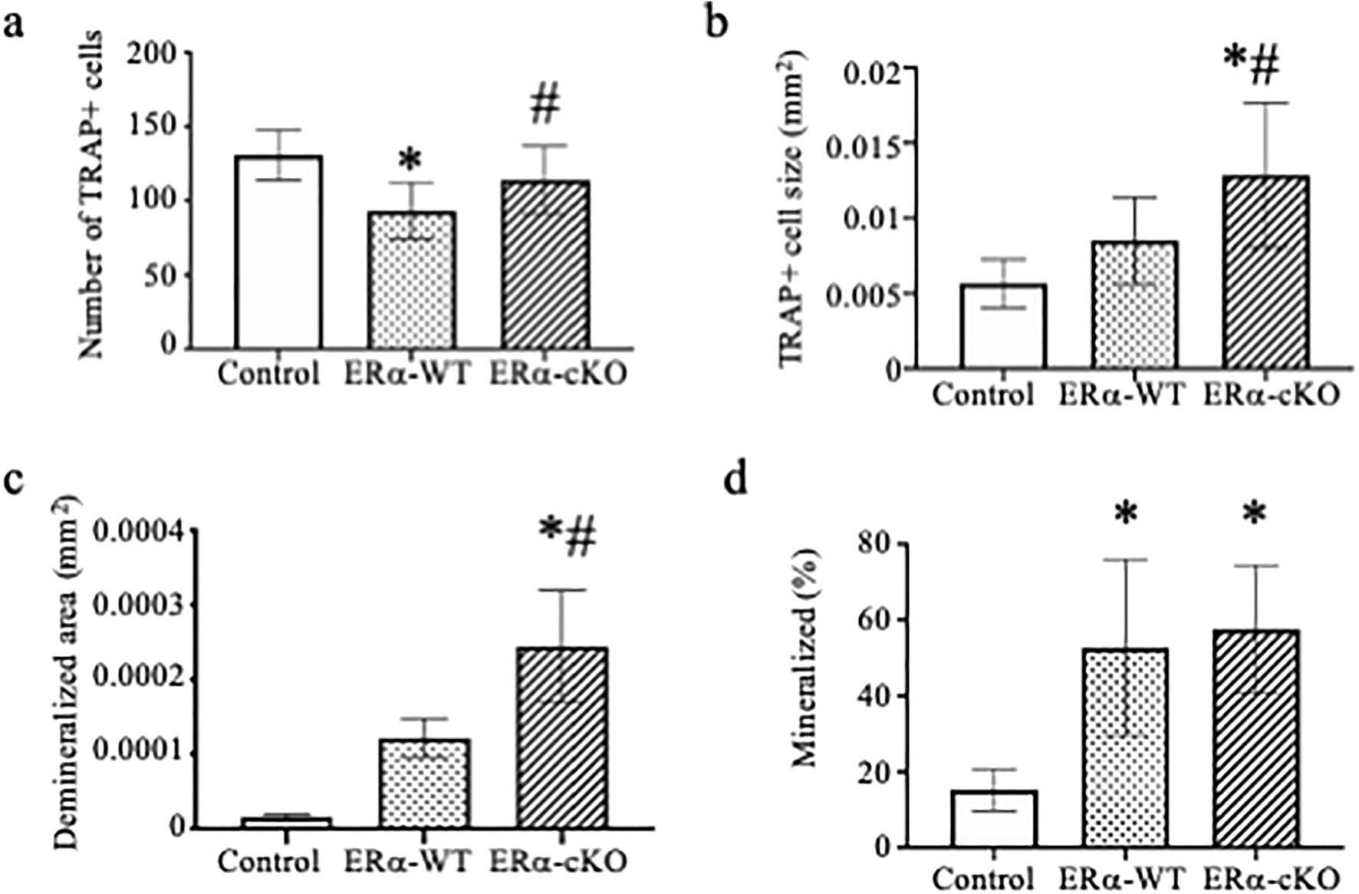
Conditioned media from estrogen receptor deficient muscle increases osteoclast differentiation. (A-C) Bone marrow macrophages were treated with M-CSF and RANKL to induce osteoclast differentiation. At the time of RANKL addition cultures were also given 5% Krebs (Control) or muscle conditioned media from ERα-WT or ERα-cKO animals. (**A**) Average number and (**B**) average size of TRAP positive multinuclear cells and (**C**) average size of demineralized area. (**D**) MC3T3-E1 cells were grown to confluency and then induced to differentiate with the addition of ascorbic acid. Krebs (Control) or muscle conditioned media from ERα-WT or ERα-cKO animals was given at the same time as ascorbic acid. Von Kossa assay was performed to measure percent mineralization of MC3T3 cell treated with 5% Krebs (control) or ERα-WT or ERα-cKO muscle conditioned media. * Significantly different from Control; ^#^Significantly different from ERα-WT.

Because several hormones are altered following the removal of ovaries, we also treated osteoclast cultures with muscle conditioned media from mice conditionally deleted for *Esr1* using the *Hsa-cre*, to determine if estrogen signaling through the major estrogen receptor was driving the responses. *Hsa-cre* is a transgenic mouse strain that targets CRE recombinase to striated muscle fibers using the human α-skeletal actin promoter^20^. Muscle contraction-induced injury, as measured by the loss of peak isometric force after ten eccentric contractions, was affected by the condition (interaction *p* < 0.001). That is, ERα-cKO muscles generated 17 and 61% less force pre- and post-injury compared to ERα-WT muscles, respectively (Fig. 2B). However, peak isometric force was reduced from pre- to post-eccentric contractions in both groups (*p* < 0.001). EDL muscle mass did not differ between ERα-cKO and ERα-WT mice (11.1 ± 1.6 and 11.4 ± 1.5 mg, respectively; *p* = 0.734). This data indicates that myokines were secreted from comparable sizes of tissue in the experiment. Osteoclast cultures treated with conditioned media from ERα-cKO (*Esr1 *^*fl/fl*^*;Hsa-cre*^+^) EDL muscle had greater cell number and size compared to those treated with media from ERα-WT (*Esr1 *^*fl/fl*^*;Hsa-cre*^*-*^) muscles (Fig. 4A, *p* = 0.012; 4B, *p* = 0.001). Osteoclasts treated with conditioned media from ERα-cKO mice also demineralized 2-fold more than osteoclasts treated with conditioned media from ERα-WT mice (Fig. 4C, *p* ≤ 0.005). Lastly, we measured MC3T3-E1 cell mineralization and conditioned media from ERα-WT and ERα-cKO animals enhanced mineralization over the control media but did not differ between media from muscles with and without ERα (Fig. 4D, *p* = 0.855).

While estrogen deficiency was previously shown to affect muscle mass and function^21^, it is unclear how perturbed estrogen-estrogen receptor signaling specific to muscle can alter myokine expression and which myokines are affected. As we had measured changes to osteoclast differentiation and activity with the addition of conditioned media from muscle lacking estrogen receptors (ERα-cKO animals) as well as animals deficient in estrogen (Figs. 3, 4), we wanted to determine if deficits of estrogen signaling specifically in muscle leads to differential production of myokines. Thus, a RT^2^ Profiler PCR array was used on ERα-WT and ERα-cKO muscle extracts to identify differences in cytokine and chemokine expression. Of the 84 test genes analyzed, 3 genes, *bone morphogenetic protein 2* (*Bmp2)*, *oncostatin M* (*Osm)* and *hemolytic complement* (*Hc*)were downregulated in the muscle from ERα-cKO relative to ERα-WT (Table 1, *p* > 0.05). There were 10 genes, *C-X-C motif chemokine ligand 11 (Cxcl11), Fas ligand (Fasl), Interferon alpha 2 (Ifnα2), Interferon gamma (Ifnγ), interleukin 12 (Il2), interleukin 22 (Il22),interleukin 15 (Il5), interleukin 7 (Il7), tumor necrosis family ligand superfamily member 11 (Tnfsf11), vascular endothelial growth factor A* (*Vegfa)* which were upregulated in the muscle from ERα-cKO relative to ER*α-*WT (Table 2, *p* > 0.05).

**Table 1:** Down regulated genes in ERα-KO muscle.

Gene	Expression in ERα-WT	Expression in ERα-KO	Fold change
*Bmp2*	1.406599	0.002652	− 530.34
*Hc*	0.307325	0.004596	− 66.87
*Osm*	0.765464	0.003384	− 226.22

**Table 2:** Up regulated genes in ERα-KO muscle.

Gene	Expression in ERα-WT	Expression in ERα-KO	Fold change
*Cxclll*	0.004645	0.010743	2.31
*Fasl*	0.004407	0.012693	2.88
*Ifnα2*	0.005046	0.012326	2.44
*Ifnγ*	0.004021	0.008221	2.04
*Il2*	0.004589	0.009343	2.04
*Il22*	0.005209	0.011539	2.22
*Ill5*	0.003264	0.009532	2.92
*Ill7*	0.006709	0.022069	3.29
*Tnfsfll(Rankl)*	0.005285	0.012891	2.44
*Vegfa*	0.203699	0.439804	2.16

**Figure 5:**
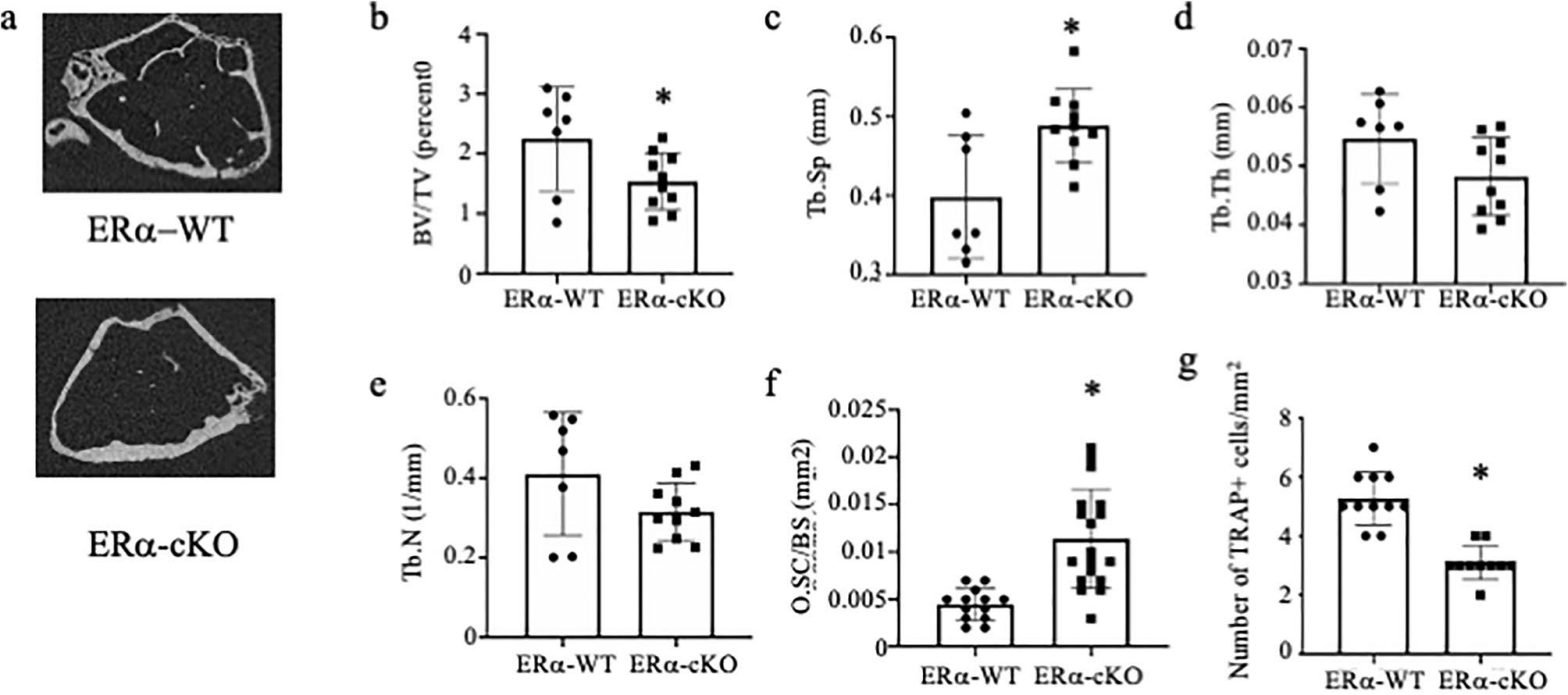
Female ERα-cKO mice are osteopenic at 9 months of age. (**A**) Representative images of trabecular bone in the distal metaphysis of femur (**B**–**E**) Trabecular micro-CT measurements of femur (**F**–**G**) histological measurements of (**F**) osteoclast surface area to bone surface and (**G**) osteoclast number/mm^2^ *Significantly different from ERα-WT.

To determine if there are in vivo differences between the skeletal system of the ERα-WT and ERα-cKO mice, we performed micro-CT analysis on femurs (Fig. 5A). ERα-cKO were osteopenic compared to their ERα-WT littermates with analysis of trabeculae within the metaphysis showing a significant decrease in bone volume to total volume ratio and an increase in trabecular spacing (Fig. 5B *p* = 0.044 and Fig. 5C *p* = 0.008). We did not measure any significant difference in trabecular number or thickness (Fig. 5D, *p* = 0.082 and Fig. 5E *p* = 0.108, respectively). Osteoclast surface area per bone surface was significantly greater in ERα-cKO bones versus ERα-WT bones (Fig. 5F, *p* ≤ 0.0001); however, number of osteoclasts per bone surface was significantly decreased in ERα-cKO compared to ERα-WT (Fig. 5G, *p* ≤ 0.0001). We found no significant difference in cortical thickness (Sup Fig. 1).

**Figure 6:**
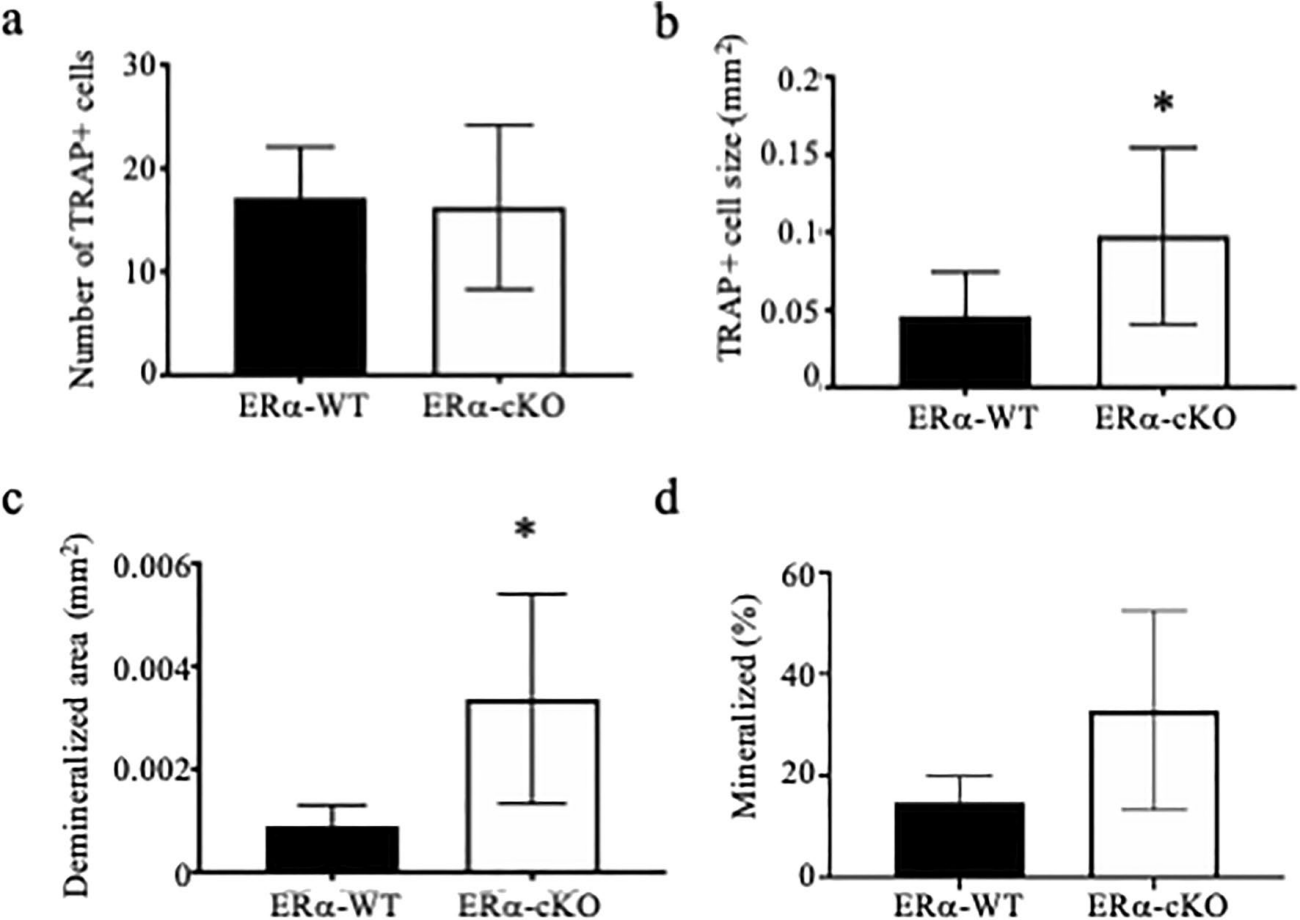
ERα-cKO mice have enhanced osteoclasts. (**A**–**C**) Bone marrow macrophages from ERα-WT and ERα-cKO animals were treated with M-CSF and RANKL to induce osteoclast differentiation. (**A**) average number and (**B**) average size of TRAP positive multinuclear cells and (**C**) average size of demineralized area. (**D**) Mesenchymal bone marrow cells were grown to confluency and induced to differentiate with the addition of ascorbic acid. Von Kossa assay was performed to measure percent mineralization of mesenchymal cells from ERα-WT and ERα-cKO animals. *Significantly different from ERα-WT.

To begin to understand which skeletal cells are contributing to the osteopenic phenotype, we cultured osteoclasts and mesenchymal stem cells from the bone marrow of ERα-WT and ERα-cKO mice. While there was no difference in the number of osteoclasts (Fig. 6A, *p* = 0.785), osteoclasts were twofold larger from bones of ERα-cKO mice (Fig. 6B, *p* = 0.040) and had larger demineralized area (Fig. 6C, *p* = 0.047). There appeared to be a greater percent of mineralization of the mesenchymal cells from the ERα-cKO mice; however, it did not reach statistical significance (Fig. 6D, *p* = 0.070). Even though *Hsa-Cre* is muscle specific, we did confirm that there were no significant changes in *Esr1* expression in mesenchymal cells and mature osteoclasts (Sup. Fig. 2).

## Discussion

Osteoporosis leads to loss of bone mass and increased risk of falls and fractures. This poses a significant health concern that affects millions worldwide^22,23^. Many studies suggest that muscle-cross talk is important for the maintenance and function of bone^2,24,25^. It is unclear how deficits in estrogen impact molecular coordination between skeletal muscle and bone and if changes in muscle serve as a potential mechanism for osteoporosis. Understanding how estrogen dynamics influence the crosstalk in the bone-muscle unit may lead to a clearer understanding of how musculoskeletal diseases develop and progress.

In our present study we determined that conditioned media from muscle enhanced both osteoclast and osteoblast differentiation. For the osteoblast experiments with the muscle conditioned media, we used an osteoblastic cell line MC3T3-E1 cells. Muscle conditioned media did enhance the ability of MC3T3-E1 cells to mineralize (Fig. 1). Muscle has been reported to secrete myokines such as insulin growth factor 1 (IGF-1), fibroblast growth factor 2 (FGF2) and IL-6 which all promote bone formation^26,27^. However, for our osteoclast experiments, we used bone marrow macrophages which were induced to form osteoclasts in the presence of macrophage colony stimulating factor (M-CSF) and receptor activator of NF-κB ligand (RANKL). We did not select or sort osteoclast precursors from the bone marrow so we cannot eliminate the possibility that there are other cells in our osteoclast cultures that responded to the myokines present in the muscle conditioned media. Future studies will need to determine if there are changes in composition, proliferation, or apoptosis of the osteoclast precursors due to treatment with muscle conditioned media. There have only been two previous studies demonstrating that muscle myokines influence osteoclast differentiation. One study demonstrated that myostatin enhances RANKL-mediated osteoclast differentiation^6^. The study went further to demonstrate that inflammatory cytokines such as interleukin 1 (IL-1), interleukin 17 (IL-17) and tumor necrosis factor alpha (TNF-α) expressed during rheumatoid arthritis leads to increases in myostatin expression which in turns enhances osteoclast differentiation^6^. The second study tested conditioned media from unloaded or loaded C2C12 cells or a cell line that is a subclone of myoblasts^11^. C2C12 conditioned media from unloaded cells inhibited osteoclast differentiation from bone marrow cultures; however, loaded C2C12 conditioned media enhanced osteoclast differentiation through expression of IL-6^11^. As far as we are aware, ours is the first study to demonstrate that myokines isolated from primary muscle regulate osteoclast differentiation.

To test if changes in estrogen signaling regulate this enhancement of osteoclast and osteoblast differentiation, we tested conditioned media from muscle of OVX mice as well as muscle from mice conditionally deleted for ERα in skeletal muscle. OVX media enhanced osteoclast differentiation but not osteoblast differentiation above sham-operated conditioned media. Similarly conditioned media from ERα-cKO mice enhanced osteoclast differentiation and activity. These data indicate the estrogen-to-estrogen receptor signaling in muscle affects bone cells. Furthermore, analyzing the skeleton of female ERα-cKO mice demonstrated that the mice are osteopenic due to an increase in osteoclast differentiation and activity. Conditioned media obtained from muscles undergoing a rigorous contraction protocol contains myokines. A PCR array was used in the present study to determine if ERα deficiency in muscle alters the myokine profile. The PCR array allowed us to analyze 84 myokine genes to determine if there is differential expression due to estrogen signaling in myocytes. ERα-cKO muscle had lower isometric force compared to ERα-WT muscle; however, both produced high force that substantially decreased after the eccentric protocol indicating high contractile activity to secrete myokines and unlikely affecting differences in cytokine expression measured. The expression of several genes was found to be down regulated including *Bmp2*, *Osm*, and *Hc* in muscle lacking ERα. These cytokines are predominantly thought to enhance bone formation^28–33^. However, in our study, muscle conditioned media from OVX and ERα-cKO mice did not enhance mineralization by MC3T3-E1 cells compared to sham or ERα-WT mice (Figs. 3, 4). Because we did not measure in vivo bone formation rate we cannot completely exclude that the altered bone phenotype we observed in the ERα-cKO mice was not due to changes in osteoblast activity (Fig. 6).

Additionally, several cytokines that promote osteoclast differentiation were upregulated in muscle from ERα-cKO mice such as *Ifn-γ, Il-22*, *Il-15* and *Il-17* and *Rankl*. We measured increased expression of cytokine genes that are known to enhance osteoclast differentiation in pathological conditions in muscle lacking ERα, however, the magnitude of the upregulated genes was quite small relative to downregulated genes. Several interferon and interleukin genes were modestly upregulated ~ twofold. IFN-γ has dual actions on osteoclast differentiation by inhibiting osteoclast differentiation through direct stimulation but enhancing osteoclast differentiation through stimulating T cells secretion of RANKL and TNF-α^34^. IL-17 stimulates osteoblasts to produce pro-osteoclastogenic cytokines such as IL-6, TNF-α and RANKL, and it has been suggested that IL-17 mediates bone loss during estrogen deficient conditions^35,36^. IL-22 and IL-15 have also been shown to promote osteoclast differentiation. IL-22 in pathological conditions such as rheumatoid arthritis by upregulating RANKL expression and IL-15 synergistically enhancing RANKL induced osteoclast differentiation^37,38^. Most of the upregulated cytokines identified affect osteoclast differentiation by modulating the expression levels of RANKL. The changes in RANKL levels induced by the myokines may explain why we measured changes in osteoclast size as increasing levels of RANKL may enhance fusion. These data suggest that the changes we observed in in vivo osteoclast differentiation and activity in the ERα-cKO mice may be due to a combination of changes in cytokine expression and not just change in one cytokine.

We were able to measure increased osteoclast differentiation when we flushed bone marrow from ERα-cKO compared to ERα-WT mice (Fig. 6). This data suggests that myokines may target osteoclast progenitors or target the bone marrow population such that we would measure changes in osteoclast differentiation in the absence of their bone environment. Loss of IL-15 expression has shown to decrease the number CFU-GM and CFU-M colonies in the tibia^39^. Additionally, a study with the myokine irisin demonstrated that muscle overexpression of irisin in a mouse model enhanced osteoclast differentiation by recruiting more osteoclast progenitors^40^. This data may suggest that changes in myokine signaling alters composition or recruitment of osteoclast progenitors.

In conclusion, our results indicate that changes in signaling between estrogen and ERα in muscle results in changes in myokine expression that are capable of altering bone cells. These changes in myokine expression suggest another mechanism by which estrogen deficiency contributes to the coupling of bone and muscle loss. Future studies will need to confirm changes in expression of specific cytokines that are driving the phenotype of the ERα-cKO mice.

## Material and methods

### Ethics

The use and care of the mice was reviewed and approved by the University of Minnesota Institutional Animal Care and Use Committee, IACUC protocol number 1907-37248A. All methods were done in accordance with appropriate guidelines and regulations. Euthanasia was performed by overdose of sodium pentobarbital (200 mg/kg body mass). Femora and tibiae were harvested following euthanasia. A piece of the tail was cut to verify the genotype of each transgenic mouse. Study was done in accordance with the ARRIVE guidelines.

### Mice

Mice were housed in groups of 4–5 and had access to phytoestrogen-free rodent chow (Harlan-Teklad #2019; Indianapolis, IN) and water ad libitum. The housing room was maintained on a 14:10 h light: dark cycle with controlled temperature and humidity. Female C57BL/6 J mice aged 3–5 month were obtained from Jackson Laboratories (Bar, ME) for the initial conditioned media experiments and the ovariectomy studies. Prior to any surgery, mice received extended-release buprenorphine subcutaneously (1.0 mg/kg body mass) as an analgesic. Mice were then anesthetized using an induction chamber containing isoflurane and then maintained using inhalation of 1.5% isoflurane in oxygen at a flow rate of 100–200 ml/min. Bilateral ovariectomy was performed through two small dorsal incisions between the iliac crest and the lower ribs^41^. Sham mice underwent the same procedures except that the ovaries were not excised. In vitro muscle experiments occurred 2–3 mo after sham or ovariectomy surgeries.

HSA-Cre (strain #0061490) were obtained from Jackson Laboratory (Bar Harbor, ME). ERα-floxed mice were previously described^42^. To generate skeletal muscle specific knockout of ERα, the breeding scheme as described in Collins et al. was followed^43^. Female skeletal muscle specific estrogen receptor α knock-out mice (*Esr1*^*fl/fl*^*;Hsa-Cre*^+^*;* ERα-cKO, N = 10) and their female wildtype littermates (*Esr1*^*fl/fl*^*;Hsa-Cre*^-^; ERα-WT, N = 7) were used in experiments at 8–10 mo of age^43^.

### In vitro muscle physiology and myokine secretion

Mice were anesthetized with pentobarbital (75 mg/kg body mass) with supplemental doses given as needed. Extensor digitorum longus (EDL) muscles were dissected and mounted in a chamber containing 1.50 mL of Krebs–Ringer bicarbonate buffer (in mM; 144 Na^+^, 126.5 Cl^−^, 6 K^+^, 1 Mg^2+^, 1 SO_4_^2−^, 1 PO_4_^3−^, 25 HCO_3_^−^, 1.25 Ca^2+^, 10 glucose, and 0.10 U/ml insulin). The buffer was equilibrated with 95% O_2_-5% CO_2_ gas and maintained at 25 °C. The distal and proximal tendons were attached by 5–0 silk suture and cyanoacrylate adhesive to a fixed support and lever arm of a servomotor system (300B-LR; Aurora Scientific Inc.; Aurora, ON, Canada), respectively. Muscles were maintained at a resting tension of 0.40 g. After a 10 min equilibrium period, isometric tetanic contractions (200 ms trains of 0.2 ms pulses at 175 Hz with 150 V) were initiated every 2 min until a plateau in peak tetanic force was obtained (pre-injury isometric force). Resting tension of 0.40 g was re-established by adjusting muscle length. This optimal muscle length (L_o_) was measured from myotendinous junction to myotendinous junction. Ten maximal eccentric contractions were then performed (injury protocol). We chose this contraction-induced injury protocol because of the high forces generated by eccentric contractions, theoretically enhancing the release of myokines. For each eccentric contraction, the muscle was passively shortened to 85% L_o_ and then stimulated for 600 ms while the muscle was simultaneously lengthened to 115% L_o_ (i.e., 30% muscle length change) at 0.5 L_o_/s. Two minutes following the last eccentric contraction, peak tetanic force was re-measured (post-injury isometric force). Krebs–Ringer bicarbonate buffer was collected separately for each individual mouse, flash frozen and stored at − 80 °C until used in conditioned media experiments.

### In vitro bone analyses

#### Primary osteoclast cell culture

Bone marrow was harvested from femora and tibiae of female C57/BL/6 J mice and non-adherent cells were differentiated into osteoclasts as done previously with minor modifications^44^. Beginning two days after re-plating bone marrow macrophages (BMMs, referred to as day 0), cells were fed 1% CMG 14–12 supernatant (Dr. Sunao Takeshita, Nagoya City University, Nagoya, Japan) which contains M-CSF and 10 ng/ml RANKL (R&D Systems) every 48 h (on days 0, 2, 4) until the desired experimental end point of TRAP^+^ cells with > 3 nuclei. Cells were plated at 40,000 cells/cm^2^.

#### Tartrate-resistant acid phosphatase (TRAP) staining

Osteoclasts were cultured to the desired time point and fixed in 4% paraformaldehyde for 20 min at 4 °C and then washed in PBS. TRAP activity was stained using Naphthol AS-MX phosphate and Fast Violet LB salt. Cells were imaged using cellSens software (Olympus) and analyzed using NIH ImageJ.

#### Measure of osteoclast demineralization

Mononuclear BMMs were plated on 24-well Corning Osteoassay surface plates at a density of 100,000 cells per well and fed as described above. At day 4–6 cells were washed away with 10% bleach as per manufacturer’s recommendations. Wells were imaged by light microscopy, and demineralization measurements were determined using NIH ImageJ.

#### Isolation of bone marrow mesenchymal cells

Adherent cells from bone marrow of female ERα-WT and ERα-cKO mice were grown in alpha-MEM without ascorbic acid (Gibco catalog# A10490) and 10% FBS (Atlanta Biologicals) 25 units/mL penicillin/streptomycin (Invitrogen), 400 mM L-Glutamine (Invitrogen). Cells were plated at a density of 52,000 cells/cm^2^. After 3 days, cells were spilt into 24 well dishes and treated with alpha-MEM media containing 1ug/mL ascorbic acid and dexamethasone for 14 days. Cells were given ascorbic acid, dexamethasone and β-glycerophosphate to induce mineralization two days before von Kossa staining. Von Kossa staining was performed, cells were photographed using cellSens software and quantitated using NIH ImageJ.

#### RNA extraction and analysis

RNA was isolated from cells plated in triplicate using Trizol reagent (Ambion, Life Technologies) and quantified using UV spectroscopy. 1 μg of RNA was used to synthesize cDNA with the iScript cDNA synthesis kit (Bio-Rad) as per the manufacturer’s protocol. Quantitative real-time PCR (RT-qPCR) was performed using the MyiQ Single Color Real-Time PCR Detection System (Bio-Rad). Each 20 μl reaction contained 1 ul of cDNA, 10 μl of iTaq Universal SYBR Green Supermix, and 25 μM forward and reverse primers. The PCR conditions were as follows: 95 °C for 3 min, and the 40 cycles of 94 °C for 15 s, 58 °C for 30 s and 72 °C for 30 s, followed by melting curve analysis (95 °C for 5 s, 65 °C for 5 s and then 65 °C to 95 °C with 0.5 °C increase every 5 s). Experimental genes were normalized to *Hprt*. All measurements were performed in triplicate and analyzed using the ΔΔCT method. *Hprt* F GAG GAG TCC TGT TGA TGT TGC CAG; *Hprt* R GGC TGG CCT ATA GGC TCA TAG TGC; *Esr1* F ACC ATT GAC AAG AAC CGG AG; and *Esr1* R CCT GAA GCA CCC ATT TCA TT.

#### Conditioned media experiments with MC3T3 and Von Kossa Staining

MC3T3-E1 cells were grown and transferred to 24 well plates and grown to confluency in alpha-MEM without ascorbic acid, 10% FBS, 25 units/mL penicillin/streptomycin, 400 mM L-Glutamine. Cells were plated at a density of 53,000 cells/cm^2^. MC3T3-E1 cells were treated with 5% Krebs, conditioned media from muscle of ERα-WT, ERα-KO mice, sham-operated or ovariectomized mice. MC3T3-E1 cells were fed with conditioned media from individual mice, alpha-MEM, and 1ug/mL ascorbic acid every 3 days for 11 days. MC3T3-E1 cells were obtained from ATCC and maintained under recommended conditions. On the 11th day the cells were fed with conditioned media, alpha-MEM, ascorbic acid and inorganic phosphate overnight. The following day von Kossa staining was performed, cells were photographed, and mineralization was quantitated using NIH ImageJ. Cells were plated in triplicate. Experiments were done with conditioned media from at least 3 individual mice.

#### Conditioned media experiments with osteoclasts

Osteoclasts from the bone marrow of female mice were generated as described above. At day 0 when bone marrow macrophages were treated with RANKL and CMG 14–12 conditioned media containing M-CSF, cells were also treated with 5% Krebs, conditioned media from the muscle of individual ERα-WT, ERα-cKO, sham-operated or ovariectomized mice until multinuclear cells appeared. Cells were TRAP stained, imaged, and size and number were quantified using NIH Image J. Experiments were done with at least 3 different mice of each genotype or condition in triplicate.

#### RT Profiler PCR array of potential myokines.

Tibialis anterior muscles from ERα-WT and ERα-cKO mice were isolated. Each muscle was placed in a safe lock 1.5 ml Eppendorf tube with 1.0 mm and 2.0 mm Zirconium oxide beads and 300 µl of TRIzol. Muscles were homogenized in a Bullet Blender for 5 min. RNA isolation was performed using TRIzol (Ambion, Life Technologies) per manufacturer's instructions and quantified using UV spectroscopy. RNA was used to synthesize cDNA with the iScript cDNA synthesis kit (Bio-Rad) as per the manufacturer’s protocol. The RT^2^ Profiler PCR array measures the expression of 84 chemokines and cytokines (mouse cytokines and chemokines; Qiagen Cat. no. 330231 PAMM-150ZA). Samples were run for one 10 min cycle at 95 °C, then 40 cycles alternating between 15 s at 95 °C and 1 min at 60 °C. ERα-WT and ERα-cKO data were analyzed on Gene Globe. Eighty-four test genes were evaluated by PCR array. All 84 test genes on the PCR array were compared to *B2m* and *Hsp90ab1*. For PCR array data analysis, a 2-fold change cutoff criterion was used to determine if the gene was upregulated or down regulated.

### In vivo skeletal analyses

#### Sample harvest

Femora and tibiae were de-fleshed and for each female mouse the right femur and tibiae were immediately stored in PBS and frozen without fixation for micro-computed tomography (micro-CT). The left tibiae were fixed in Z-fix (Anatech LTD) and decalcified in 10% EDTA (pH 7.4) for paraffin-embedded sectioning and histological staining. The left femur was used to harvest bone marrow-derived macrophages (BMMs) for in vitro culture.

#### Staining of paraffin-embedded sections

Decalcified bone sections were de-paraffinized in xylene, rehydrated through an ethanol gradient, and stained for TRAP at 37 °C for 1 h as described above. Sections were then counterstained with methyl-green for 15 s, cover slipped was placed using Permount mounting media (Electron Microscopy Sciences) and allowed to rest for 24 h before imaging.

#### Micro-CT Analysis

Frozen right femora were equilibrated to room temperature and scanned in PBS with a 1 mm aluminum filter using the XT H 225 micro-computed tomography machine (Nikon Metrology Inc., Brighton, MI, USA) at an isotropic voxel size of 7.4 µm. The scan settings were 120 kV, 61 µA, 720 projections, 2 frames per projection, and an integration time of 708 ms. CT Pro 3D (Nikon metrology, Inc., Brighton, MI, USA) was used to make 3D reconstruction volumes for each scan. VGStudio MAX 3.2 (Volume Graphics GmbH, Heidelberg, Germany) was used to convert 3D reconstruction volumes to bitmap datasets for each scan. Morphometric analysis was completed with the SkyScan CT-Analyser (CTAn, Bruker micro-CT, Belgium) following Bruker’s instructions and reported guidelines for the field^45^. The region of interest for trabecular bone analysis in the distal metaphysis started 0.5 mm proximal to the growth plate and extended 1.5 mm proximally towards the diaphysis. The region for cortical bone analysis was a 0.5 mm region at the mid-diaphysis. Automated contouring was used to determine the region of interest boundaries for both trabecular and cortical bone with manual editing as needed. Global thresholding was used to segment bone from surrounding tissue for both 3D trabecular and 2D cortical analyses. One threshold value was used for all cortical analyses, and a different threshold value was used for all trabecular analyses. CT-Volume (Bruker micro-CT, Belgium) was used to create all 3D models from bitmaps corresponding to cortical and trabecular regions analyzed; however, only a 1 mm region of the most distal trabecular selection was used to create a model. Genotype of mice was not known until all micro-CT analysis was done so that results could be grouped together.

### Statistical testing

All data are presented as means with standard deviations. Data presented in graphs for in vitro bone cell experiments represents an average of at least three biologically independent experiments done in triplicate performed with either MC3T3-E1 or bone marrow cells from independent mice. In vivo data represents all samples harvested for that specific experiment graphed together. No samples were removed as outliers in any experiment. For the bone cell measurements, t-tests were used to compare two groups and one-way ANOVAs with Tukey’s multiple comparisons test used when comparing three groups. To analyze EDL peak isometric tetanic force before and after the bout of eccentric contractions, two-way AVOVAs were used (time [pre vs post] by condition [Sham vs OVX] or [ERα-WT vs ERα-cKO) with Holm-Sidak post-hoc tests. Significance was set at *p* < 0.05. Statistical testing was performed in GraphPad Prism 8 or SigmaPlot version 12.0.

## Supplementary Information

Supplementary Figure 1.

Supplementary Figure 2.

## Data Availability

The datasets generated during and/or analyzed during the current study are available from the corresponding author on reasonable request.
