# Supplementary figures and images for: Estrogen regulation of myokines that enhance osteoclast differentiation and activity
Source: Sci Rep. 2022 Sep 23;12:15900. doi: 10.1038/s41598-022-19438-4 (PMC9508086; doi:10.1038/s41598-022-19438-4)

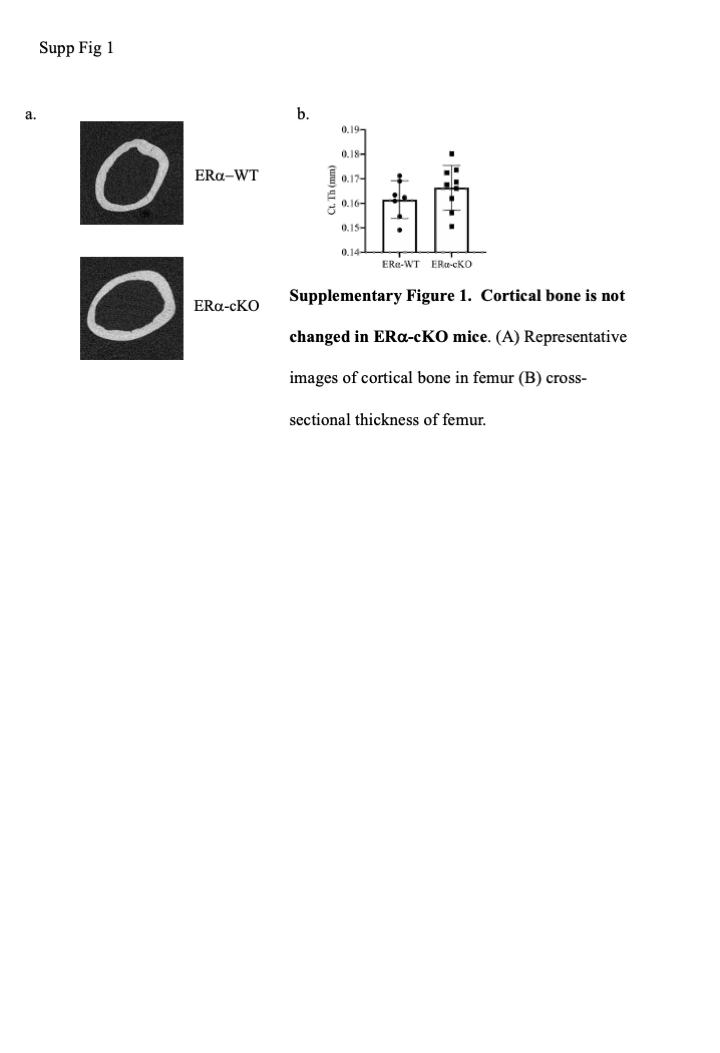

Supplement: Supplementary file 1 — Supplementary Figure 1. [file 41598_2022_19438_MOESM1_ESM.tiff]

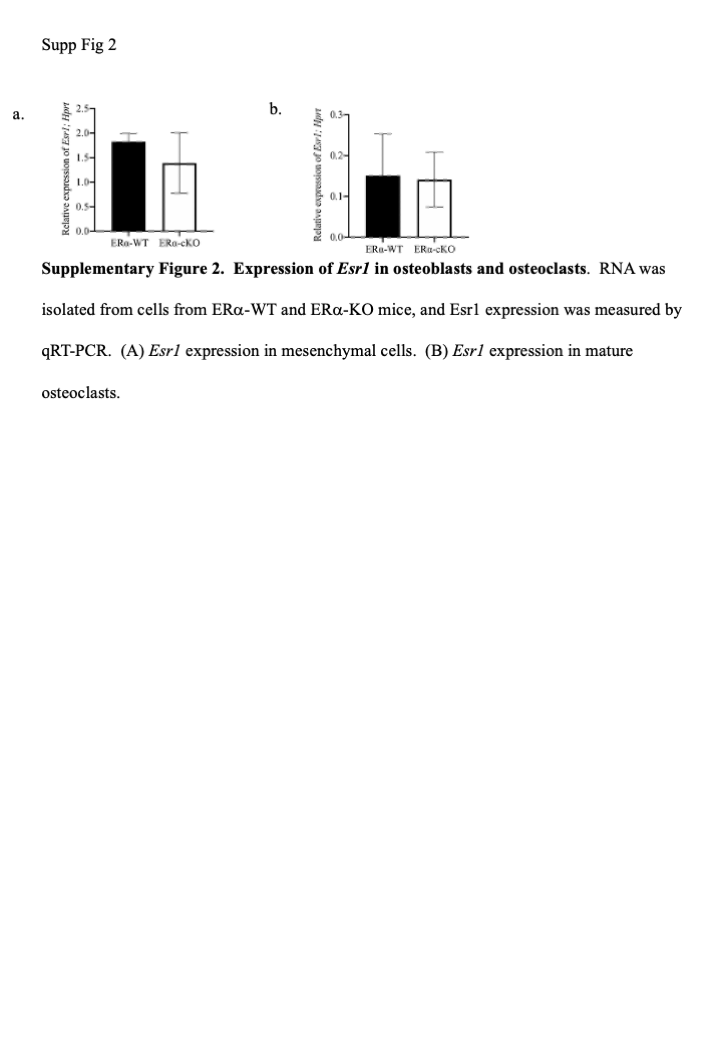

Supplement: Supplementary file 2 — Supplementary Figure 2. [file 41598_2022_19438_MOESM2_ESM.tiff]
